# Nonunion with Breakage of Gamma Nail and Subsequent Fracture in the Ipsilateral Femur

**DOI:** 10.1155/2013/534570

**Published:** 2013-02-21

**Authors:** Takahiro Niikura, Sang Yang Lee, Yoshitada Sakai, Kotaro Nishida, Ryosuke Kuroda, Masahiro Kurosaka

**Affiliations:** Department of Orthopaedic Surgery, Kobe University Graduate School of Medicine, 7-5-1 Kusunoki-cho, Chuo-ku, Kobe 650-0017, Japan

## Abstract

We describe a rare case with breakage of gamma nail accompanied by nonunion of the original fracture and a subsequent new fracture in the ipsilateral femur. A 73-year-old woman suffered a subtrochanteric fracture of the femur, and the fracture was fixed with gamma nail at a previous hospital. However, fracture reduction was not adequately achieved and a large gap remained between the fracture fragments. The fracture demonstrated atrophic nonunion 10 months after surgery, and autologous bone grafting was performed at the same hospital. Two months after the second surgery, a breakage of the nail at the distal screw hole was observed. Twenty-six months after the second surgery, the patient fell and a fracture occurred at the level of the nail breakage. The atrophic nonunion site and fresh fracture site were very close thus demonstrating a segmental fracture. We exchanged the original gamma nail with a long gamma nail and performed autologous bone grafting at the nonunion site. Both the fresh fracture site and the nonunion site obtained bony union. This tragic chain of events was caused by inappropriate initial treatment and replacing the nail to a longer nail and autologous bone grafting were effective as salvage surgery.

## 1. Introduction

Gamma nail is a commonly used implant for the treatment of proximal femur fractures [1] and breakage of the nail is a relatively rare complication. We present a rare patient with breakage of gamma nail accompanied by nonunion of the original fracture and a subsequent new fracture in the ipsilateral femur. We describe the case and discuss the causes and treatment.

## 2. Case Report

**Figure 1:**
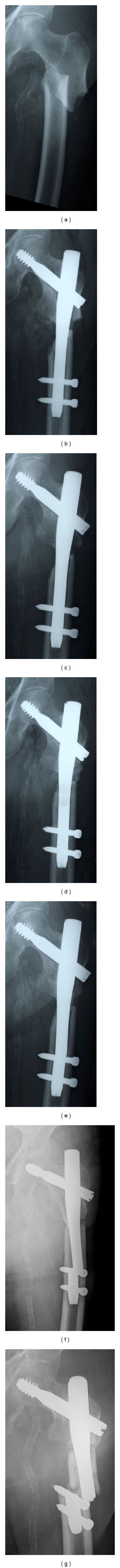
Consecutive history before surgery at our hospital. (a) Subtrochanteric fracture, (b) initial surgery, (c) nonunion at 10 months after surgery, (d) autologous bone grafting, (e) nail breakage at 2 months after bone grafting, (f) 24 months after bone grafting, (g) fracture at nail breakage 26 months after bone grafting.

The patient gave the informed consent prior being included into this case report. A 73-year-old woman suffered a subtrochanteric fracture of the left femur (Figure 1(a)). She had accompanying chronic renal failure and had been treated by hemodialysis for 40 years. The fracture was fixed with a second-generation gamma nail (Gamma APJ (Asia Pacific Japan) nail, Stryker, Japan) at a previous hospital; however, fracture reduction was not adequately achieved and a large gap remained between the fracture fragments (Figure 1(b)). Ten months after the initial surgery, the gap was still evident demonstrating atrophic nonunion (Figure 1(c)). Autologous bone grafting to the nonunion site was performed at the same hospital (Figure 1(d)). Cancellous bone was harvested from the iliac crest and grafted. Two months after the second surgery, a breakage of the nail at the distal screw hole was observed and the grafted bone appeared to be absorbed (Figure 1(e)). However the patient refused further surgery, and X-ray followup was continued up to 24 months after the second surgery (Figure 1(f)).

**Figure 2:**
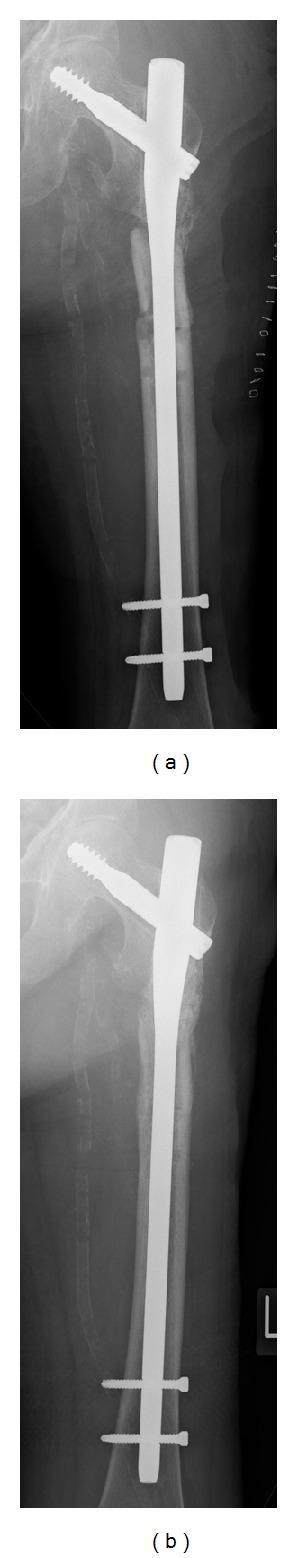
After surgery at our hospital. (a) Replacing the nail with the longer nail supplemented with autologous bone grafting at the long-standing nonunion site. (b) Bony union of both the older and newer fracture.

Twenty-six months after the second surgery (36 months after initial surgery), the patient fell and a fracture occurred at the level of the nail breakage, and she was referred to our hospital. The atrophic nonunion site and fresh fracture site were very close thus demonstrating a segmental fracture (Figure 1(g)). We exchanged the original gamma nail with a long gamma nail (Long Gamma APJ nail, Stryker, Japan), and performed autologous bone grafting at the nonunion site (Figure 2(a)). Cancellous bone was harvested from the iliac crest and grafted. The fresh fracture site healed uneventfully six months after the surgery, additionally the nonunion site obtained bony union nine months after the surgery (Figure 2(b)). At the last followup 15 months postoperatively, the patient could walk with the aid of a walker and was pain-free. Further followup could not be performed due to her death.

## 3. Discussion

Breakage of a gamma nail is a relatively rare complication, and the reported incidence ranges from 0.2 to 5.7% [2–9]. The location of the nail breakage was reported mainly at the level of the aperture of the lag screw and at the distal screw hole, followed by the nail shaft [3–9]. In our case the nail breakage occurred at the distal screw hole.

The tragic chain of events in this case was caused by previous inappropriate surgery, which apparently caused the nail breakage. At the initial surgery, fracture reduction was not obtained and a large gap continued to exist between the fragments, however, the insertion of the nail was performed and interlocked with two distal screws. There was no bony contact between the fragments. The previous surgeon then performed autologous bone grafting to the fracture gap at the second surgery without revision of the implant. However replacement of the gamma nail was indicated when making attempts at gap reduction during the second surgery. Moreover, a long gamma nail was also indicated in the initial surgery because the fracture was in the subtrochanteric area [5, 7, 10–12]. No bony contact led to excessive stress to the hardware. Weight bearing was transmitted from the proximal fragment directly to the nail and the distal locking screw. Therefore, we suggest the nail broke due to metal fatigue at the location with the strongest stress.

Replacing the nail and autologous bone grafting was effective for salvage surgery. A longer nail was selected to fix the segmental fracture. A long gamma nail is commonly used for treatment of subtrochanteric fracture and its effectiveness has been reported [5, 7, 10–12]. Additionally, Barquet et al. reported a good clinical result in the treatment of subtrochanteric nonunion with long gamma nails [13]. We predicted the fresh fracture would heal by fixation; however, the nonunion site located above the fresh fracture site was long standing and thought to be biologically less active. Therefore, we supplemented with autologous bone grafting at the nonunion site.

Patient-dependent risk factors for nonunion include various medical comorbidities (e.g., diabetes, vascular disease), advanced age, smoking, alcohol abuse, nonsteroidal anti-inflammatory drug use, nutritional deficiency, radiation treatment, genetic disorders, and metabolic disease or endocrine pathology [14]. This patient had accompanying chronic renal failure and had been treated by hemodialysis for 40 years. There is no comprehensive study describing the relationship between nonunion and chronic renal failure/hemodialysis. However, Karaeminogullari et al. reported the high incidence of nonunion in surgically treated hip fractures in patients undergoing chronic hemodialysis [15]. We cannot declare that the chronic renal failure/hemodialysis impaired the fracture healing, however, it is possible that the chronic renal failure/hemodialysis affected this patient's fracture healing. This patient was 73-year-old; therefore the advanced age possibly affected the impaired fracture healing.

## 4. Conclusion

We present a rare patient with breakage of gamma nail accompanied by nonunion of the original fracture and a subsequent new fracture in the ipsilateral femur. This case confirms our understanding that appropriate reduction and fixation is important in fracture surgery. Replacing the nail to a longer nail and autologous bone grafting were effective as salvage surgery.
